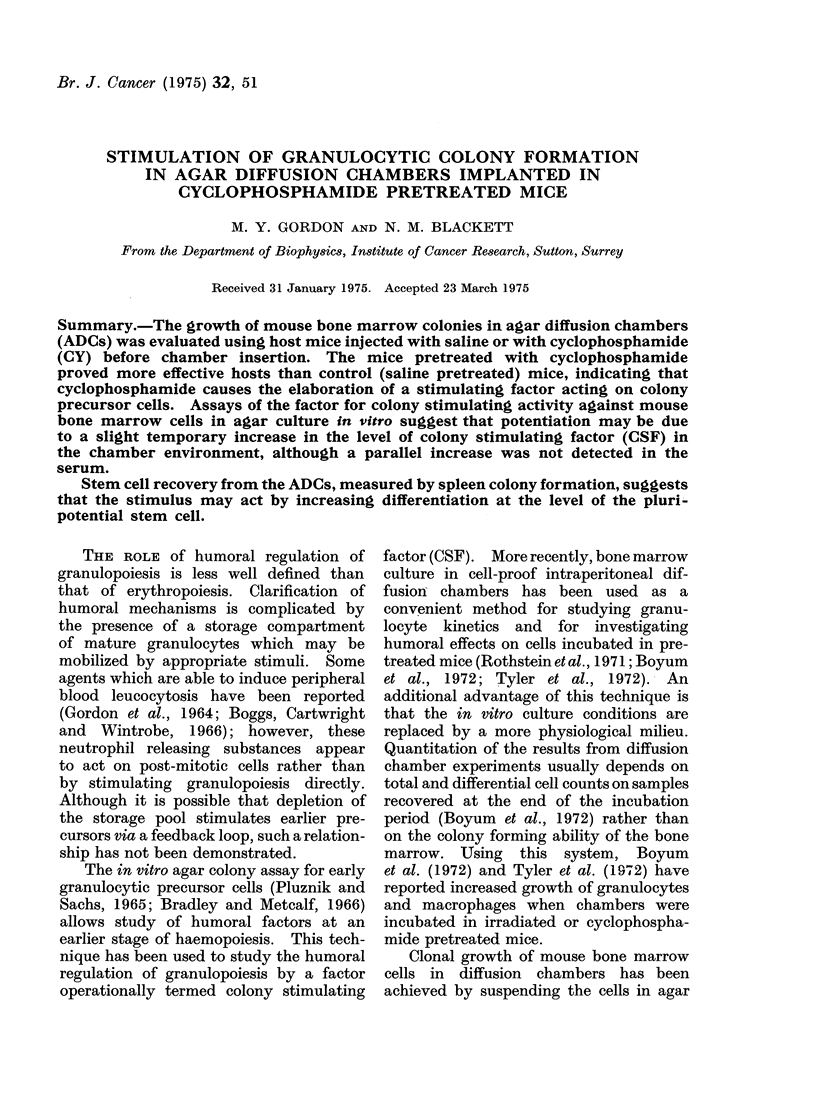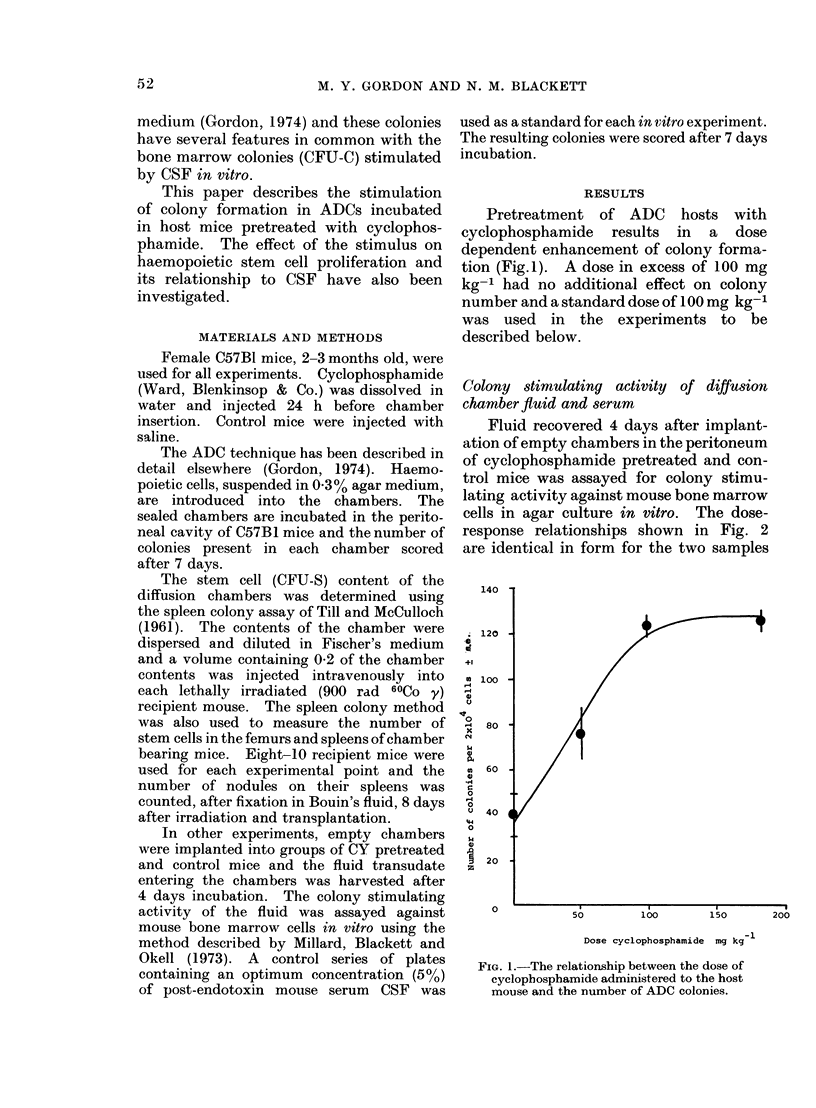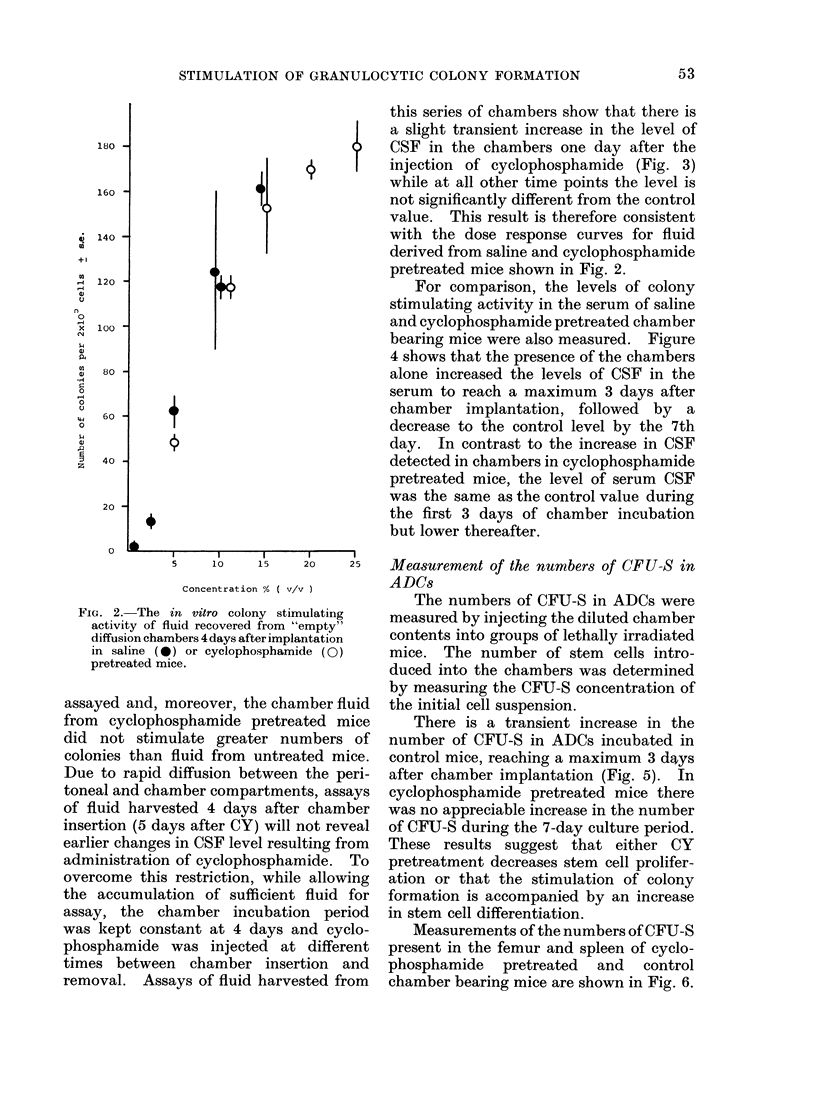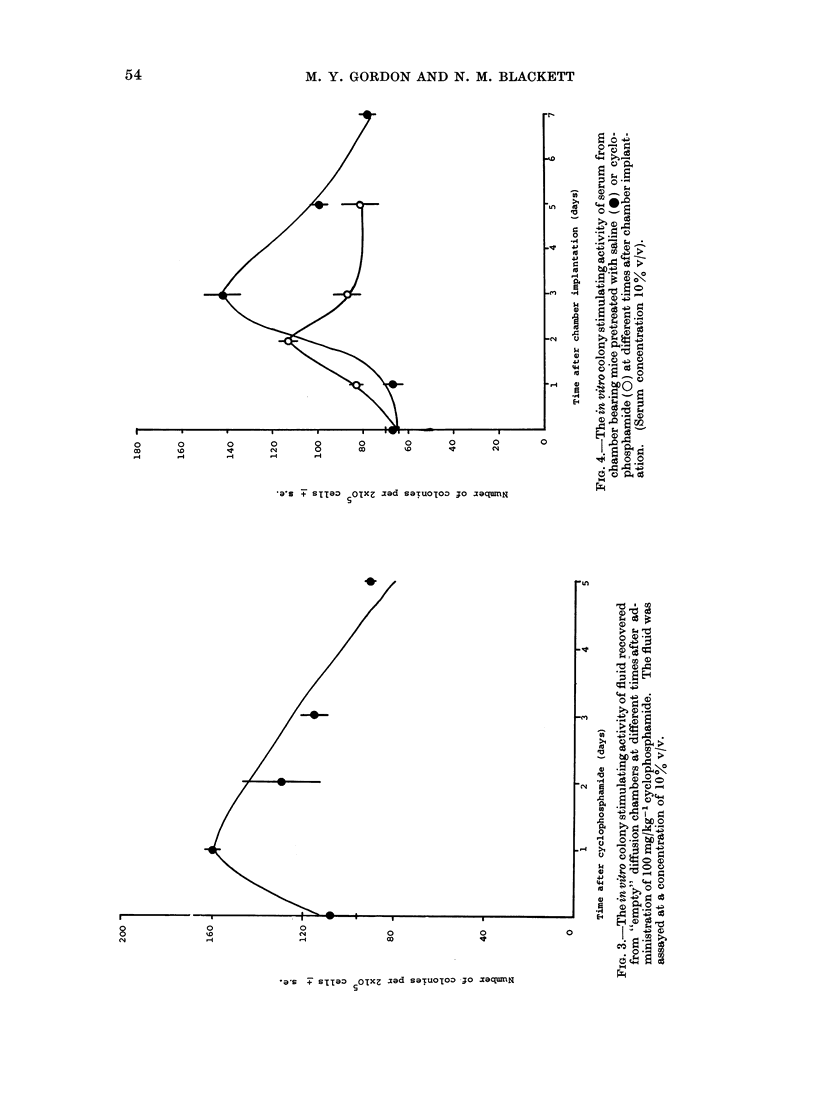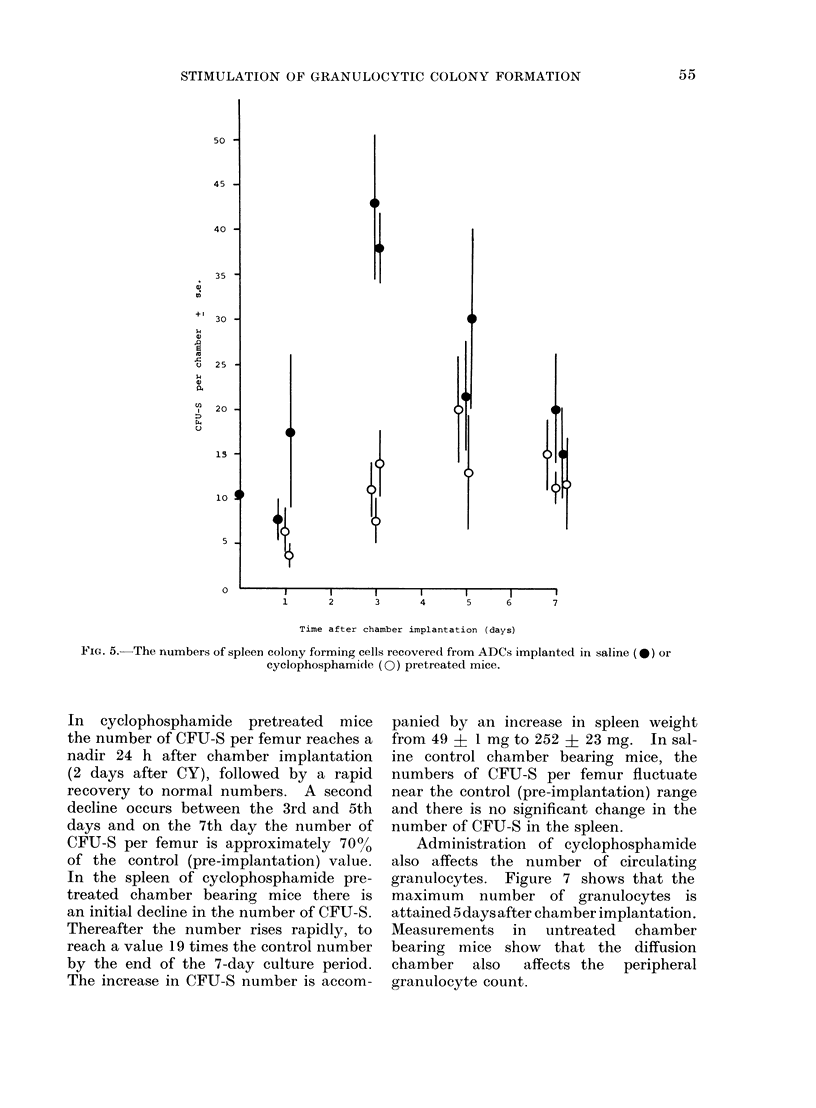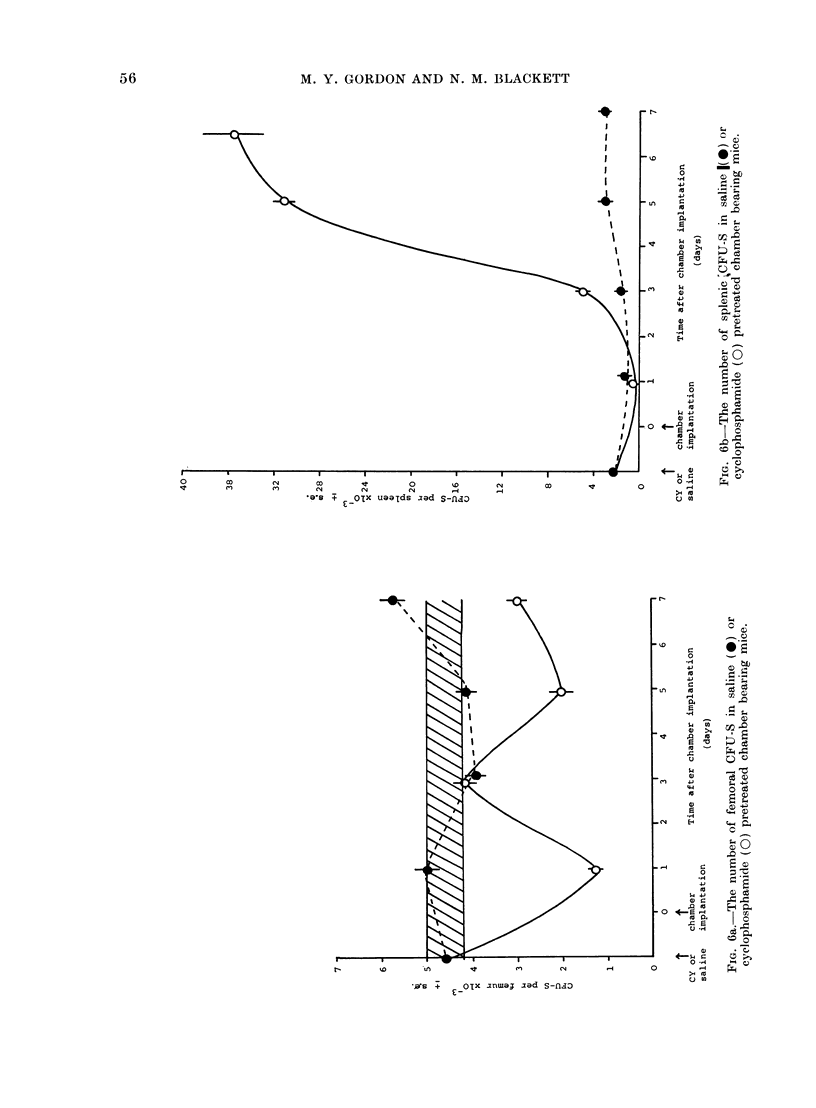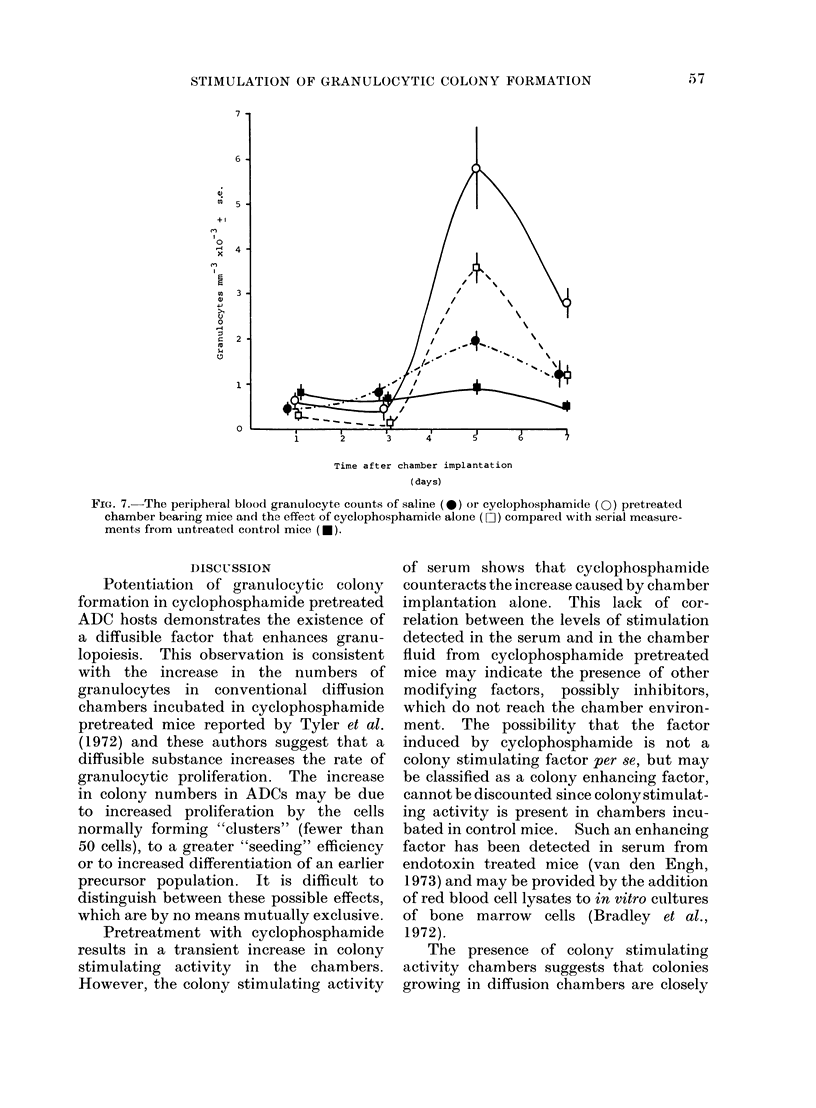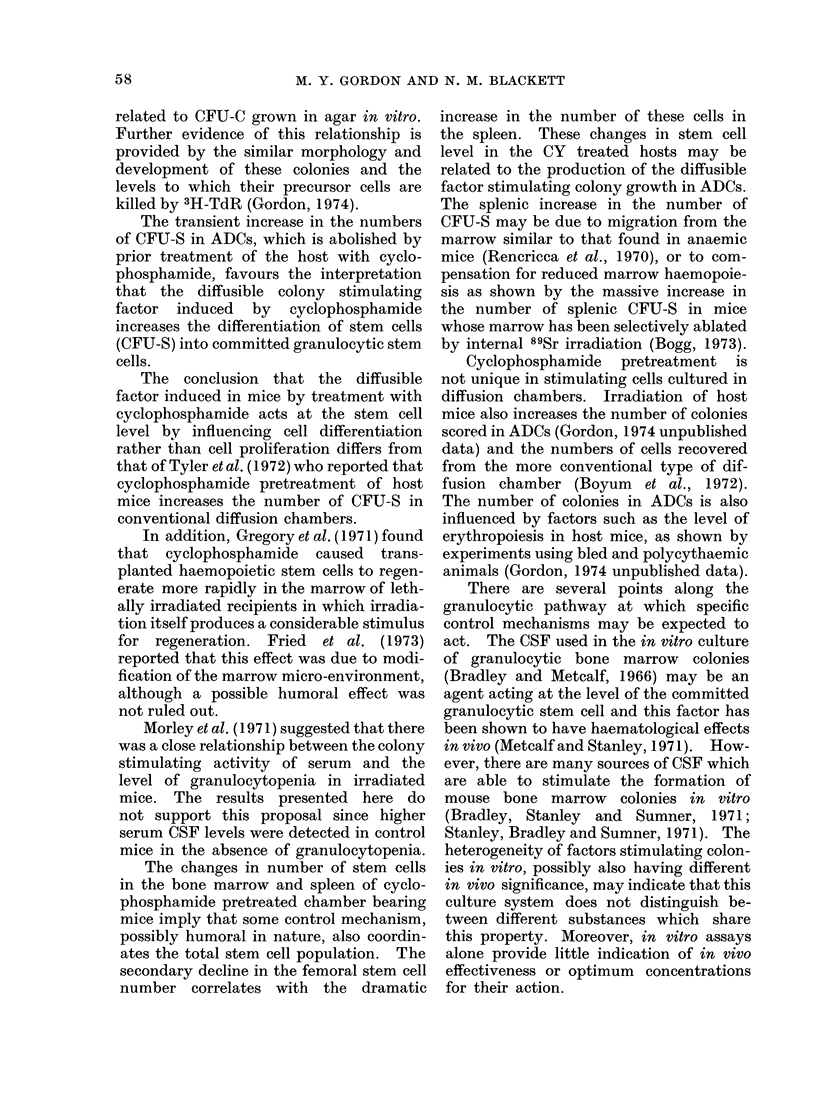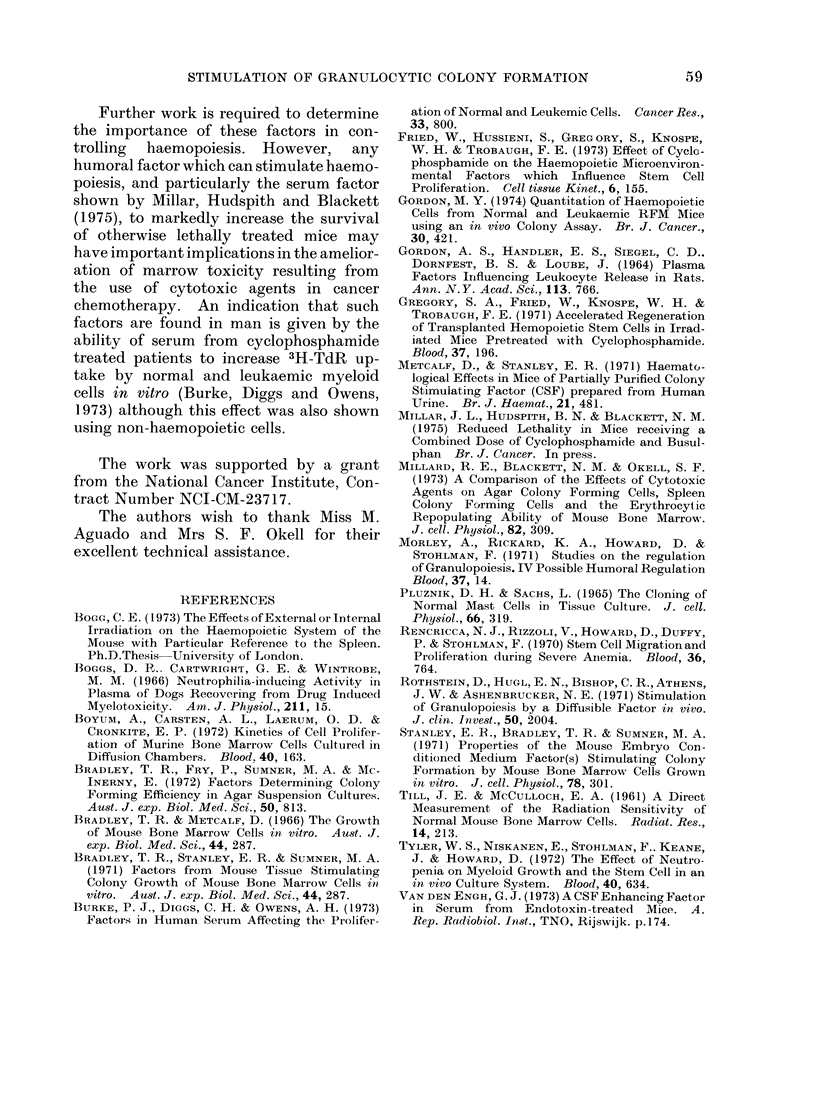# Stimulation of granulocytic colony formation in agar diffusion chambers implanted in cyclophosphamide pretreated mice.

**DOI:** 10.1038/bjc.1975.133

**Published:** 1975-07

**Authors:** M. Y. Gordon, N. M. Blackett

## Abstract

The growth of mouse bone marrow colonies in agar diffusion chambers (ADCs) was evaluated using host mice injected with saline or with cyclophosphamide (CY) before chamber insertion. The mice pretreated with cyclophosphamide proved more effective hosts than control (saline pretreated) mice, indicating that cyclophosphamide causes the elaboration of a stimulating factor acting on colony precursor cells. Assays of the factor for colony stimulating activity against mouse bone marrow cells in agar culture in vitro suggest that potentiation may be due to a slight temporary increase in the level of colony stimulating factor (CSF) in the chamber environment, although a parallel increase was not detected in the serum. Stem cell recovery from the ADCs, measured by spleen colony formation, suggests that the stimulus may act by increasing differentiation at the level of the pluripotential stem cell.


*Br. J. Cancer* (1975) **32**, 51

### STIMULATION OF GRANULOCYTIC COLONY FORMATION IN AGAR DIFFUSION CHAMBERS IMPLANTED IN CYCLOPHOSPHAMIDE PRETREATED MICE

M. Y. GORDON AND N. M. BLACKETT

*From the Department of Biophysics, Institute of Cancer Research, Sutton, Surrey*

Received 31 January 1975. Accepted 23 March 1975

**Summary.—The growth of mouse bone marrow colonies in agar diffusion chambers (ADCs) was evaluated using host mice injected with saline or with cyclophosphamide (CY) before chamber insertion. The mice pretreated with cyclophosphamide proved more effective hosts than control (saline pretreated) mice, indicating that cyclophosphamide causes the elaboration of a stimulating factor acting on colony precursor cells. Assays of the factor for colony stimulating activity against mouse bone marrow cells in agar culture *in vitro* suggest that potentiation may be due to a slight temporary increase in the level of colony stimulating factor (CSF) in the chamber environment, although a parallel increase was not detected in the serum.**

**Stem cell recovery from the ADCs, measured by spleen colony formation, suggests that the stimulus may act by increasing differentiation at the level of the pluripotential stem cell.**

THE ROLE of humoral regulation of granulopoiesis is less well defined than that of erythropoiesis. Clarification of humoral mechanisms is complicated by the presence of a storage compartment of mature granulocytes which may be mobilized by appropriate stimuli. Some agents which are able to induce peripheral blood leucocytosis have been reported (Gordon *et al.*, 1964; Boggs, Cartwright and Wintrobe, 1966); however, these neutrophil releasing substances appear to act on post-mitotic cells rather than by stimulating granulopoiesis directly. Although it is possible that depletion of the storage pool stimulates earlier precursors *via* a feedback loop, such a relationship has not been demonstrated.

The *in vitro* agar colony assay for early granulocytic precursor cells (Pluznik and Sachs, 1965; Bradley and Metcalf, 1966) allows study of humoral factors at an earlier stage of haemopoiesis. This technique has been used to study the humoral regulation of granulopoiesis by a factor operationally termed colony stimulating factor (CSF). More recently, bone marrow culture in cell-proof intraperitoneal diffusion chambers has been used as a convenient method for studying granulocyte kinetics and for investigating humoral effects on cells incubated in pretreated mice (Rothstein *et al.*, 1971; Boyum *et al.*, 1972; Tyler *et al.*, 1972). An additional advantage of this technique is that the *in vitro* culture conditions are replaced by a more physiological milieu. Quantitation of the results from diffusion chamber experiments usually depends on total and differential cell counts on samples recovered at the end of the incubation period (Boyum *et al.*, 1972) rather than on the colony forming ability of the bone marrow. Using this system, Boyum *et al.* (1972) and Tyler *et al.* (1972) have reported increased growth of granulocytes and macrophages when chambers were incubated in irradiated or cyclophosphamide pretreated mice.

Clonal growth of mouse bone marrow cells in diffusion chambers has been achieved by suspending the cells in agar medium (Gordon, 1974) and these colonies have several features in common with the bone marrow colonies (CFU-C) stimulated by CSF *in vitro*.

This paper describes the stimulation of colony formation in ADCs incubated in host mice pretreated with cyclophosphamide. The effect of the stimulus on haemopoietic stem cell proliferation and its relationship to CSF have also been investigated.

### MATERIALS AND METHODS

Female C57Bl mice, 2–3 months old, were used for all experiments. Cyclophosphamide (Ward, Blenkinsop & Co.) was dissolved in water and injected 24 h before chamber insertion. Control mice were injected with saline.

The ADC technique has been described in detail elsewhere (Gordon, 1974). Haemopoietic cells, suspended in 0·3% agar medium, are introduced into the chambers. The sealed chambers are incubated in the peritoneal cavity of C57Bl mice and the number of colonies present in each chamber scored after 7 days.

The stem cell (CFU-S) content of the diffusion chambers was determined using the spleen colony assay of Till and McCulloch (1961). The contents of the chamber were dispersed and diluted in Fischer's medium and a volume containing 0·2 of the chamber contents was injected intravenously into each lethally irradiated (900 rad $^{60}$Co $\gamma$) recipient mouse. The spleen colony method was also used to measure the number of stem cells in the femurs and spleens of chamber bearing mice. Eight–10 recipient mice were used for each experimental point and the number of nodules on their spleens was counted, after fixation in Bouin's fluid, 8 days after irradiation and transplantation.

In other experiments, empty chambers were implanted into groups of CY pretreated and control mice and the fluid transudate entering the chambers was harvested after 4 days incubation. The colony stimulating activity of the fluid was assayed against mouse bone marrow cells *in vitro* using the method described by Millard, Blackett and Okell (1973). A control series of plates containing an optimum concentration (5%) of post-endotoxin mouse serum CSF was used as a standard for each *in vitro* experiment. The resulting colonies were scored after 7 days incubation.

### RESULTS

Pretreatment of ADC hosts with cyclophosphamide results in a dose dependent enhancement of colony formation (Fig.1). A dose in excess of 100 mg kg$^{-1}$ had no additional effect on colony number and a standard dose of 100 mg kg$^{-1}$ was used in the experiments to be described below.

### *Colony stimulating activity of diffusion chamber fluid and serum*

Fluid recovered 4 days after implantation of empty chambers in the peritoneum of cyclophosphamide pretreated and control mice was assayed for colony stimulating activity against mouse bone marrow cells in agar culture *in vitro*. The dose-response relationships shown in Fig. 2 are identical in form for the two samples

FIG. 1.—The relationship between the dose of cyclophosphamide administered to the host mouse and the number of ADC colonies.

Fig. 2.—The *in vitro* colony stimulating activity of fluid recovered from "empty" diffusion chambers 4 days after implantation in saline (●) or cyclophosphamide (○) pretreated mice.

assayed and, moreover, the chamber fluid from cyclophosphamide pretreated mice did not stimulate greater numbers of colonies than fluid from untreated mice. Due to rapid diffusion between the peritoneal and chamber compartments, assays of fluid harvested 4 days after chamber insertion (5 days after CY) will not reveal earlier changes in CSF level resulting from administration of cyclophosphamide. To overcome this restriction, while allowing the accumulation of sufficient fluid for assay, the chamber incubation period was kept constant at 4 days and cyclophosphamide was injected at different times between chamber insertion and removal. Assays of fluid harvested from this series of chambers show that there is a slight transient increase in the level of CSF in the chambers one day after the injection of cyclophosphamide (Fig. 3) while at all other time points the level is not significantly different from the control value. This result is therefore consistent with the dose response curves for fluid derived from saline and cyclophosphamide pretreated mice shown in Fig. 2.

For comparison, the levels of colony stimulating activity in the serum of saline and cyclophosphamide pretreated chamber bearing mice were also measured. Figure 4 shows that the presence of the chambers alone increased the levels of CSF in the serum to reach a maximum 3 days after chamber implantation, followed by a decrease to the control level by the 7th day. In contrast to the increase in CSF detected in chambers in cyclophosphamide pretreated mice, the level of serum CSF was the same as the control value during the first 3 days of chamber incubation but lower thereafter.

*Measurement of the numbers of CFU-S in ADCs*

The numbers of CFU-S in ADCs were measured by injecting the diluted chamber contents into groups of lethally irradiated mice. The number of stem cells introduced into the chambers was determined by measuring the CFU-S concentration of the initial cell suspension.

There is a transient increase in the number of CFU-S in ADCs incubated in control mice, reaching a maximum 3 days after chamber implantation (Fig. 5). In cyclophosphamide pretreated mice there was no appreciable increase in the number of CFU-S during the 7-day culture period. These results suggest that either CY pretreatment decreases stem cell proliferation or that the stimulation of colony formation is accompanied by an increase in stem cell differentiation.

Measurements of the numbers of CFU-S present in the femur and spleen of cyclophosphamide pretreated and control chamber bearing mice are shown in Fig. 6.

FIG. 3.—The *in vitro* colony stimulating activity of fluid recovered from "empty" diffusion chambers at different times after administration of 100 mg/kg$^{-1}$ cyclophosphamide. The fluid was assayed at a concentration of 10% v/v.

FIG. 4.—The *in vitro* colony stimulating activity of serum from chamber bearing mice pretreated with saline (●) or cyclophosphamide (○) at different times after chamber implantation. (Serum concentration 10% v/v).

FIG. 5.—The numbers of spleen colony forming cells recovered from ADCs implanted in saline (●) or cyclophosphamide (○) pretreated mice.

In cyclophosphamide pretreated mice the number of CFU-S per femur reaches a nadir 24 h after chamber implantation (2 days after CY), followed by a rapid recovery to normal numbers. A second decline occurs between the 3rd and 5th days and on the 7th day the number of CFU-S per femur is approximately 70% of the control (pre-implantation) value. In the spleen of cyclophosphamide pretreated chamber bearing mice there is an initial decline in the number of CFU-S. Thereafter the number rises rapidly, to reach a value 19 times the control number by the end of the 7-day culture period. The increase in CFU-S number is accompanied by an increase in spleen weight from 49 ± 1 mg to 252 ± 23 mg. In saline control chamber bearing mice, the numbers of CFU-S per femur fluctuate near the control (pre-implantation) range and there is no significant change in the number of CFU-S in the spleen.

Administration of cyclophosphamide also affects the number of circulating granulocytes. Figure 7 shows that the maximum number of granulocytes is attained 5 days after chamber implantation. Measurements in untreated chamber bearing mice show that the diffusion chamber also affects the peripheral granulocyte count.

FIG. 6b—The number of splenic CFU-S in saline (●) or cyclophosphamide (○) pretreated chamber bearing mice.

FIG. 6a.—The number of femoral CFU-S in saline (●) or cyclophosphamide (○) pretreated chamber bearing mice.

FIG. 7.—The peripheral blood granulocyte counts of saline (●) or cyclophosphamide (○) pretreated chamber bearing mice and the effect of cyclophosphamide alone (□) compared with serial measurements from untreated control mice (■).

DISCUSSION

Potentiation of granulocytic colony formation in cyclophosphamide pretreated ADC hosts demonstrates the existence of a diffusible factor that enhances granulopoiesis. This observation is consistent with the increase in the numbers of granulocytes in conventional diffusion chambers incubated in cyclophosphamide pretreated mice reported by Tyler *et al.* (1972) and these authors suggest that a diffusible substance increases the rate of granulocytic proliferation. The increase in colony numbers in ADCs may be due to increased proliferation by the cells normally forming "clusters" (fewer than 50 cells), to a greater "seeding" efficiency or to increased differentiation of an earlier precursor population. It is difficult to distinguish between these possible effects, which are by no means mutually exclusive.

Pretreatment with cyclophosphamide results in a transient increase in colony stimulating activity in the chambers. However, the colony stimulating activity of serum shows that cyclophosphamide counteracts the increase caused by chamber implantation alone. This lack of correlation between the levels of stimulation detected in the serum and in the chamber fluid from cyclophosphamide pretreated mice may indicate the presence of other modifying factors, possibly inhibitors, which do not reach the chamber environment. The possibility that the factor induced by cyclophosphamide is not a colony stimulating factor *per se*, but may be classified as a colony enhancing factor, cannot be discounted since colony stimulating activity is present in chambers incubated in control mice. Such an enhancing factor has been detected in serum from endotoxin treated mice (van den Engh, 1973) and may be provided by the addition of red blood cell lysates to *in vitro* cultures of bone marrow cells (Bradley *et al.*, 1972).

The presence of colony stimulating activity chambers suggests that colonies growing in diffusion chambers are closely related to CFU-C grown in agar *in vitro*. Further evidence of this relationship is provided by the similar morphology and development of these colonies and the levels to which their precursor cells are killed by $^{3}$H-TdR (Gordon, 1974).

The transient increase in the numbers of CFU-S in ADCs, which is abolished by prior treatment of the host with cyclophosphamide, favours the interpretation that the diffusible colony stimulating factor induced by cyclophosphamide increases the differentiation of stem cells (CFU-S) into committed granulocytic stem cells.

The conclusion that the diffusible factor induced in mice by treatment with cyclophosphamide acts at the stem cell level by influencing cell differentiation rather than cell proliferation differs from that of Tyler *et al.* (1972) who reported that cyclophosphamide pretreatment of host mice increases the number of CFU-S in conventional diffusion chambers.

In addition, Gregory *et al.* (1971) found that cyclophosphamide caused transplanted haemopoietic stem cells to regenerate more rapidly in the marrow of lethally irradiated recipients in which irradiation itself produces a considerable stimulus for regeneration. Fried *et al.* (1973) reported that this effect was due to modification of the marrow micro-environment, although a possible humoral effect was not ruled out.

Morley *et al.* (1971) suggested that there was a close relationship between the colony stimulating activity of serum and the level of granulocytopenia in irradiated mice. The results presented here do not support this proposal since higher serum CSF levels were detected in control mice in the absence of granulocytopenia.

The changes in number of stem cells in the bone marrow and spleen of cyclophosphamide pretreated chamber bearing mice imply that some control mechanism, possibly humoral in nature, also coordinates the total stem cell population. The secondary decline in the femoral stem cell number correlates with the dramatic increase in the number of these cells in the spleen. These changes in stem cell level in the CY treated hosts may be related to the production of the diffusible factor stimulating colony growth in ADCs. The splenic increase in the number of CFU-S may be due to migration from the marrow similar to that found in anaemic mice (Rencricca *et al.*, 1970), or to compensation for reduced marrow haemopoiesis as shown by the massive increase in the number of splenic CFU-S in mice whose marrow has been selectively ablated by internal $^{89}$Sr irradiation (Bogg, 1973).

Cyclophosphamide pretreatment is not unique in stimulating cells cultured in diffusion chambers. Irradiation of host mice also increases the number of colonies scored in ADCs (Gordon, 1974 unpublished data) and the numbers of cells recovered from the more conventional type of diffusion chamber (Boyum *et al.*, 1972). The number of colonies in ADCs is also influenced by factors such as the level of erythropoiesis in host mice, as shown by experiments using bled and polycythaemic animals (Gordon, 1974 unpublished data).

There are several points along the granulocytic pathway at which specific control mechanisms may be expected to act. The CSF used in the *in vitro* culture of granulocytic bone marrow colonies (Bradley and Metcalf, 1966) may be an agent acting at the level of the committed granulocytic stem cell and this factor has been shown to have haematological effects *in vivo* (Metcalf and Stanley, 1971). However, there are many sources of CSF which are able to stimulate the formation of mouse bone marrow colonies *in vitro* (Bradley, Stanley and Sumner, 1971; Stanley, Bradley and Sumner, 1971). The heterogeneity of factors stimulating colonies *in vitro*, possibly also having different *in vivo* significance, may indicate that this culture system does not distinguish between different substances which share this property. Moreover, *in vitro* assays alone provide little indication of *in vivo* effectiveness or optimum concentrations for their action.

Further work is required to determine the importance of these factors in controlling haemopoiesis. However, any humoral factor which can stimulate haemopoiesis, and particularly the serum factor shown by Millar, Hudspith and Blackett (1975), to markedly increase the survival of otherwise lethally treated mice may have important implications in the amelioration of marrow toxicity resulting from the use of cytotoxic agents in cancer chemotherapy. An indication that such factors are found in man is given by the ability of serum from cyclophosphamide treated patients to increase $^3$H-TdR uptake by normal and leukaemic myeloid cells *in vitro* (Burke, Diggs and Owens, 1973) although this effect was also shown using non-haemopoietic cells.

The work was supported by a grant from the National Cancer Institute, Contract Number NCI-CM-23717.

The authors wish to thank Miss M. Aguado and Mrs S. F. Okell for their excellent technical assistance.

### REFERENCES

Bogg, C. E. (1973) The Effects of External or Internal Irradiation on the Haemopoietic System of the Mouse with Particular Reference to the Spleen. Ph.D.Thesis—University of London.

Boggs, D. R., Cartwright, G. E. & Wintrobe, M. M. (1966) Neutrophilia-inducing Activity in Plasma of Dogs Recovering from Drug Induced Myelotoxicity. *Am. J. Physiol.*, **211**, 15.

Boyum, A., Carsten, A. L., Laerum, O. D. & Cronkite, E. P. (1972) Kinetics of Cell Proliferation of Murine Bone Marrow Cells Cultured in Diffusion Chambers. *Blood*, **40**, 163.

Bradley, T. R., Fry, P., Sumner, M. A. & McInerny, E. (1972) Factors Determining Colony Forming Efficiency in Agar Suspension Cultures. *Aust. J. exp. Biol. Med. Sci.*, **50**, 813.

Bradley, T. R. & Metcalf, D. (1966) The Growth of Mouse Bone Marrow Cells *in vitro*. *Aust. J. exp. Biol. Med. Sci.*, **44**, 287.

Bradley, T. R., Stanley, E. R. & Sumner, M. A. (1971) Factors from Mouse Tissue Stimulating Colony Growth of Mouse Bone Marrow Cells *in vitro*. *Aust. J. exp. Biol. Med. Sci.*, **44**, 287.

Burke, P. J., Diggs, C. H. & Owens, A. H. (1973) Factors in Human Serum Affecting the Proliferation of Normal and Leukemic Cells. *Cancer Res.*, **33**, 800.

Fried, W., Hussieni, S., Gregory, S., Knospe, W. H. & Trobaugh, F. E. (1973) Effect of Cyclophosphamide on the Haemopoietic Microenvironmental Factors which Influence Stem Cell Proliferation. *Cell tissue Kinet.*, **6**, 155.

Gordon, M. Y. (1974) Quantitation of Haemopoietic Cells from Normal and Leukaemic RFM Mice using an *in vivo* Colony Assay. *Br. J. Cancer.*, **30**, 421.

Gordon, A. S., Handler, E. S., Siegel, C. D., Dornfest, B. S. & Loube, J. (1964) Plasma Factors Influencing Leukocyte Release in Rats. *Ann. N.Y. Acad. Sci.*, **113**. 766.

Gregory, S. A., Fried, W., Knospe, W. H. & Trobaugh, F. E. (1971) Accelerated Regeneration of Transplanted Hemopoietic Stem Cells in Irradiated Mice Pretreated with Cyclophosphamide. *Blood*, **37**, 196.

Metcalf, D., & Stanley, E. R. (1971) Haematological Effects in Mice of Partially Purified Colony Stimulating Factor (CSF) prepared from Human Urine. *Br. J. Haemat.*, **21**, 481.

Millar, J. L., Hudspith, B. N. & Blackett, N. M. (1975) Reduced Lethality in Mice receiving a Combined Dose of Cyclophosphamide and Busulphan *Br. J. Cancer.* In press.

Millard, R. E., Blackett, N. M. & Okell, S. F. (1973) A Comparison of the Effects of Cytotoxic Agents on Agar Colony Forming Cells, Spleen Colony Forming Cells and the Erythrocytic Repopulating Ability of Mouse Bone Marrow. *J. cell. Physiol.*, **82**, 309.

Morley, A., Rickard, K. A., Howard, D. & Stohlman, F. (1971) Studies on the regulation of Granulopoiesis. IV Possible Humoral Regulation *Blood*, **37**, 14.

Pluznik, D. H. & Sachs, L. (1965) The Cloning of Normal Mast Cells in Tissue Culture. *J. cell. Physiol.*, **66**, 319.

Rencricca, N. J., Rizzoli, V., Howard, D., Duffy, P. & Stohlman, F. (1970) Stem Cell Migration and Proliferation during Severe Anemia. *Blood*, **36**, 764.

Rothstein, D., Hugl, E. N., Bishop, C. R., Athens, J. W. & Ashenbrucker, N. E. (1971) Stimulation of Granulopoiesis by a Diffusible Factor *in vivo*. *J. clin. Invest.*, **50**, 2004.

Stanley, E. R., Bradley, T. R. & Sumner, M. A. (1971) Properties of the Mouse Embryo Conditioned Medium Factor(s) Stimulating Colony Formation by Mouse Bone Marrow Cells Grown *in vitro*. *J. cell. Physiol.*, **78**, 301.

Till, J. E. & McCulloch, E. A. (1961) A Direct Measurement of the Radiation Sensitivity of Normal Mouse Bone Marrow Cells. *Radiat. Res.*, **14**, 213.

Tyler, W. S., Niskanen, E., Stohlman, F., Keane, J. & Howard, D. (1972) The Effect of Neutropenia on Myeloid Growth and the Stem Cell in an *in vivo* Culture System. *Blood*, **40**, 634.

Van den Engh, G. J. (1973) A CSF Enhancing Factor in Serum from Endotoxin-treated Mice. *A. Rep. Radiobiol. Inst.*, TNO, Rijswijk. p.174.